# A Novel Universal Approach for Temperature Correction on Frequency Domain Spectroscopy Curve of Transformer Polymer Insulation

**DOI:** 10.3390/polym11071126

**Published:** 2019-07-02

**Authors:** Jiefeng Liu, Xianhao Fan, Yiyi Zhang, Hanbo Zheng, Huilu Yao, Chaohai Zhang, Yubo Zhang, Dajian Li

**Affiliations:** 1 College of Electrical Engineering, Guangxi University, Nanning 530004, China; 2 School of Physical and Technology, Guangxi University, Nanning 530004, China; 3 Electric Power Research Institute of Guangxi Power Grid Co., Ltd., Nanning 530023, China

**Keywords:** transformer polymer insulation, frequency domain spectroscopy (FDS), shift factor (*α*_T_), master curve technique, temperature correction

## Abstract

It is a fact that the frequency domain spectroscopy (FDS) curve at different temperatures can be corrected by the shift factor (*α*_T_) extracted from the master curve. However, the *α*_T_ and master curve reported by previous works are distinctive due to the difference in the construction algorithm. Therefore, it is of great significance to report a universal approach for extracting *α*_T_. In this work, the unaged oil-immersed pressboards with different moisture content (*mc%*) are firstly prepared and selected as the research specimen. Then, the *α*_T_ of FDS curves on the above pressboard is extracted based upon the master curve technique. The influence mechanism under the various test temperature (*T*) and *mc%* is therefore analyzed so as to establish a universal model for predicting the *α*_T_. The present findings reveal that the *α*_T_ value extracted from FDS curves is both temperature-dependent and moisture-dependent. In addition, the predicted *α*_T_ is not only suitable for temperature correction on FDS curve of same type pressboard with different insulation conditions (moisture contents and aging degrees), but also maintains considerable accuracy when applied to different types of pressboard. Therefore, the obtained conclusions will provide a universal method for temperature correction on FDS curve of transformer polymer insulation.

## 1. Introduction

The performance of transformer oil–paper insulation is related to the stable operation of the entire power system. It is a fact that the aging condition of the polymer (paper) insulation of the energized transformer mainly determines the lifespan of the transformer oil–paper insulation system [1,2,3,4,5]. Thus, the condition evaluation of transformer polymer insulation has become a research hotspot. In contrast with the traditional method based upon chemical and electric parameters, the frequency domain spectroscopy (FDS) technique has received widespread attention, due to its advantages of being sensitive to insulation conditions and more suitable for field testing [6,7,8,9,10]. In the last decades, a large number of studies on the FDS technique were carried out [11,12,13,14,15,16] and the research findings reveal that temperature is one of the crucial factors affecting dielectric response measurements [17,18,19,20,21,22,23]. Provided that the contribution of the temperature effect on the FDS curve is ignored, the obtained response data are not reliable. The method for temperature correction on FDS curve is therefore of great significance. In other words, it is an urgent issue to report a reliable approach for temperature correction on the FDS curve.

In reviews of previous studies, it is pointed out that the temperature effect merely leads to the FDS curve moving along the frequency axis and will not alter its shape [20,21,22,23]. In addition, it is reported that the FDS curves at different test temperatures can be linked by shift factor (*α*_T_) [20,21,22,23]. The general shape of the curve is often preserved if the data is plotted on a log–log scale. This feature allows the formation of a master curve by shifting the curves along the frequency axis until they form a continuous curve at a chosen reference temperature [23], and the above scale factor is defined as the shift factor. A lot of work indicates that the shift factor extracted by the master curve technique can be used for temperature correction on FDS curve. However, the master curve and shift factor obtained by previous works are distinctive due to the difference in the construction algorithm. Therefore, it is of great significance to report a universal approach for extracting the shift factor without constructing the master curve. In view of this key issue, the author preliminarily reported a universal approach for calculating the shift factor, which is realized by establishing a functional relationship between the shift factor and various test temperatures [20]. The previous study indicates that the variation law between the shift factor and test temperature can be described by the Arrhenius equation. It is interesting to note that, as for the special operating environment of the transformer insulation system, the dominant reaction mechanism of polymer aging should be hydrolysis when referring to the Arrhenius process [24]. It seems likely that the value of the shift factor is both temperature-dependent and moisture-dependent [23]. Provided that the synergy effect produced by moisture content and temperature is ignored, the accuracy and versatility of the established model will be severely limited. Therefore, the universal model [20] for temperature correction on FDS curve is not appropriate if the synergy effect is not considered. 

Given this consideration, this paper is devoted to understanding the mechanism of the synergy effect on the *α*_T_ so as to propose a universal approach for temperature correction. In the present work, the unaged oil-immersed pressboards with different moisture content are firstly prepared under the controlled laboratory conditions. Next, the variation law among the shift factor, moisture content, and the temperature is deeply analyzed so as to establish a functional model for predicting the shift factor. The present findings point out that the shift factor corresponding to any test temperature and moisture content can be accurately predicted by the above functional model. Finally, the experiments of the temperature correction are performed so as to verify the feasibility and accuracy of the proposed approach. The conclusions reveal that the predicted shift factor is not only suitable for temperature correction on FDS curve of same type pressboard with different insulation conditions (moisture content and aging degree), but considerable accuracy is also maintained when it is applied to different types of paperboard. Therefore, the conclusions will provide a universal idea for temperature correction on FDS curve of transformer polymer insulation.

## 2. Sample Construction

In order to verify the versatility of the proposed method, two types of pressboard discs with different moisture contents were utilized for performing the variable temperature experiment and verification experiment. The diameter of the pressboard discs for both experiments were 160 mm. The details are described in Table 1. The transformer oil is the Karamay No. 25 naphthenic mineral oil and satisfies the standard of ASTM D3487-2000 (II).

**Figure 1:**
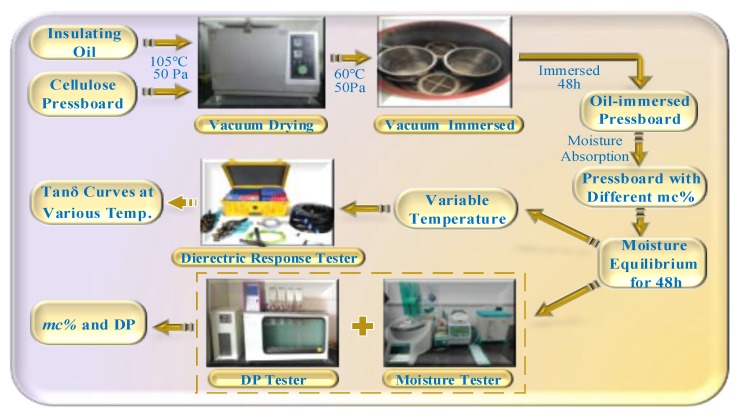
The experimental schedule of unaged pressboard with type I.

**Figure 2:**
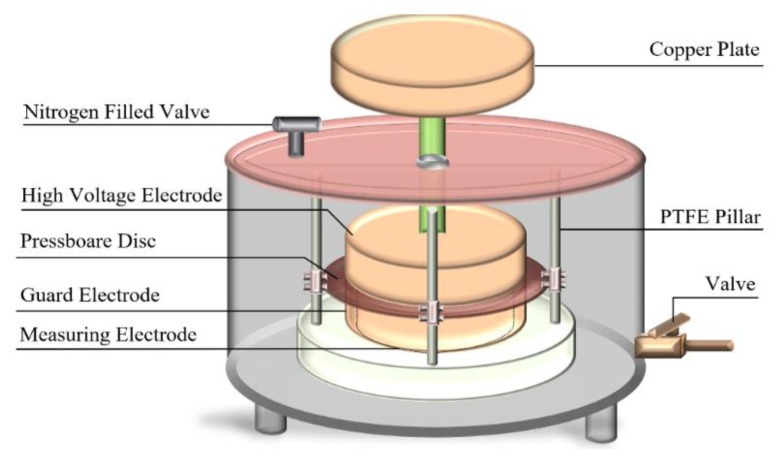
The three-electrode test cell.

As shown in Figure 1, the insulating oil and the pressboard are vacuum dried in a ratio of 20:1 at 105 °C, 50 Pa, and the oil-immersed pressboard is obtained by vacuum immersed at 60 °C, 50 Pa. The pressboard with initial moisture content *a*% is placed in a precision electronic balance and its quality (*m*) is recorded; the natural moisture absorption is later performed so as to obtain the pressboard with various expected moisture contents. Provided that the measured value of the balance reaches *m* × (1 + *b*%)/(1 + *a*%), the moisture content of the pressboard is regarded as *b*% [25,26]. The moisture balance distribution process is later performed at 45 °C, for 48 h. Subsequently, the DP value (degree of polymerization) and moisture content are obtained by a DP tester (by means of viscosity testing) and a moisture tester (by means of Karl Fischer Titration), as shown in Figure 1. In this paper, the unaged oil-immersed pressboards (type I) with different moisture contents (1.3%, 2.3%, 3.1% and 4.4%) were firstly prepared. The tan*δ* curves are tested by DIRANA, where the dielectric response measurement is achieved by three-electrode test cell, shown in Figure 2; the test voltage is set to 200 V, the test frequency is 2 × 10^−3^ − 5 × 10^3^ Hz.

## 3. The Extraction of the Shift Factor

**Figure 3:**
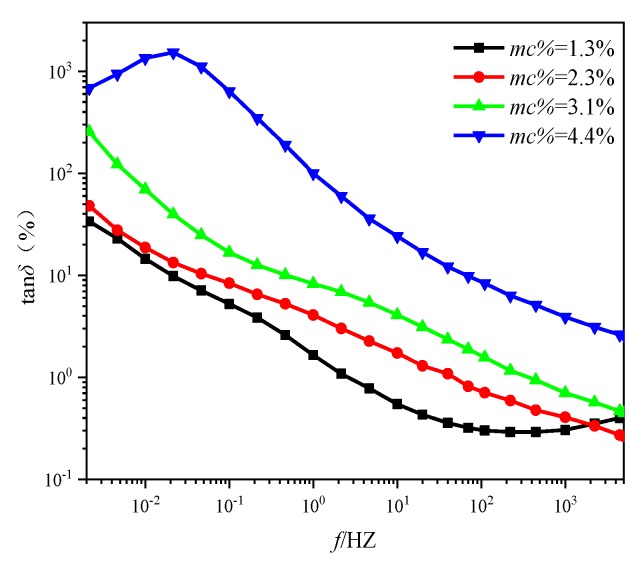
The tan*δ* curves of unaged pressboards (type I) with various *mc%* (45 °C).

**Figure 4:**
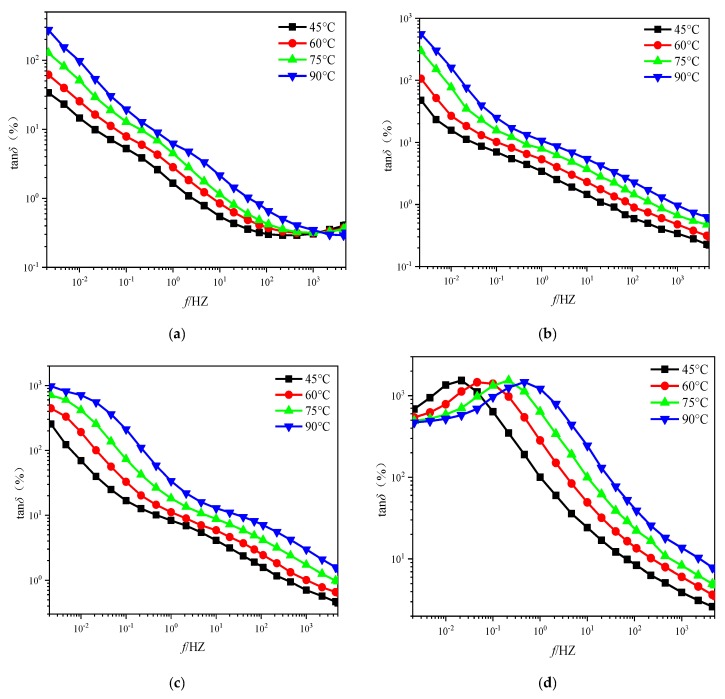
The tanδ curves of unaged pressboard with type I at various test temperatures. (**a**) *mc%* = 1.3%, (**b**) *mc%* = 2.3%, (**c**) *mc%* = 3.1%, (**d**) *mc%* = 4.4%.

In this paper, the unaged oil-immersed pressboards (type I) with various *mc%* are selected as the research object, the tan*δ* curves of above pressboards with various moisture contents are shown in Figure 3, and the test temperature is 45 °C. Then, the tan*δ* curves of the above pressboards at different test temperatures (45, 60, 75, and 90 °C) were measured, respectively, which is displayed in Figure 4.

It is a fact that the increase in ambient temperature enhances the kinetic energy of the molecules inside the pressboard samples, making it easier for the molecules to complete the orientation polarization under the action of external electric field forces. This means that the pressboard samples with the same insulation condition (aging degree and *mc%*) will need less time to establish the process of relaxation polarization under the high temperature. Therefore, the high temperature allows the relaxation polarization process to be established at a higher frequency section than the reference temperature. This interesting phenomenon can be observed on the variation law of the tan*δ* curve under the temperature effect, which is shown in Figure 4. Obviously, the high temperature will cause the tan*δ* curves to move toward the right along the frequency axis without changing its shape. This conclusion is also consistent with the existing research findings [17,18,19,20,21,22,23].

Provided that the FDS curve at different test temperatures, *T*, is moved along the frequency axis to the reference temperature (*T_ref_*) by a certain scale factor, the reference curve is defined as the master curve and the above scale factor is defined as the shift factor *α*_T_, and the formula of the shift factor is as shown in Equation (1) [20], where, *f_Tref_* and *f_T_* are the frequencies corresponding to a given point on the tan*δ* curve at the reference temperature and the test temperature, respectively. Moreover, the reference temperature in this paper is set to 45 °C.

(1) $$\alpha_{T}=\frac{f_{T}}{f_{Tref}}$$

**Figure 5:**
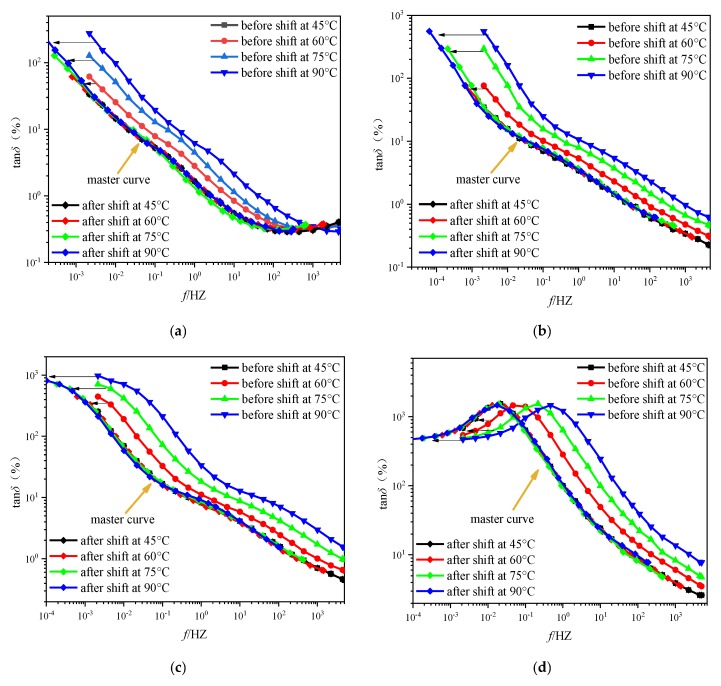
The construction results of the master curve of the unaged pressboard with type I. (**a**) *mc%* = 1.3%, (**b**) *mc%* = 2.3%, (**c**) *mc%* = 3.1%, (**d**) *mc%* = 4.4%.

As mentioned in the introduction part, the temperature effect does not alter the shape of the response curve. Consequently, the master curve obtained by shifting along the frequency axis should coincide with the measured curve at the reference temperature. The construction algorithm moves the tan*δ* curve at different test temperatures along the frequency axis to the left. In this paper, as long as the shifted curves and the measured curve at the reference temperature have the maximum coincidence degree (i.e., the sum of squared deviations reaches the minimum), the shift factor of the above test temperature is extracted immediately. Figure 5 presents the construction results.

In this paper, the fitting of the master curve is completed by least square estimation (LSE), provided that the FDS curves at reference temperature (45 °C) and various test temperatures can be represented by tan*δ*(*f_Trefi_*) and tan*δ*(*f_Ti_*), respectively. The *f_Trefi_*, *f_Ti_* represents the *i*-th sampling frequency of the tan*δ* curve at the reference temperature and test temperatures, respectively. It can be seen from Equation (1), *f_Ti_*/α_T_ = *f_Trefi_*, provided that the tan*δ* curves at different test temperatures can perfectly coincide with the master curve after translation, i.e., tan*δ*(*f_Trefi_*) = tan*δ*(*f_Ti_/*α_T_). However, the results of translation are not perfect and always bring deviations. Subsequently, the *β_i_* = [tan*δ*(*f_Trefi_*) − tan*δ*(*f_Ti_/*α_T_)]^2^ is defined as the deviation of a single sample point in the fitting process of the master curve. The following boundary conditions need to be mentioned, once (*f_Ti_/*α_T_) < min(*f_Trefi_*) or max(*f_Ti_/*α_T_) < *f_Trefi_*, the above sampling points are considered as invalid points, and the deviation *β_i_* is therefore assumed to be 0. The sum of squared deviations (∑*β_i_*) is therefore defined as the objective function of the fitting process. Once the ∑*β_i_* reaches the minimum value, the calculated α_T_ is exactly what we need. Moreover, the shift factor values of pressboard 1 extracted by the above process are presented is Table 2. It is found that the shift factor presented an increasing trend with increasing temperature and moisture content.

## 4. A Universal Model for Extracting the Shift Factor

As mentioned above, the variation law between the shift factor and test temperature can be accurately linked to the Arrhenius equation [20,21,22,23,24]. It is interesting to note that the Arrhenius equation is an empirical formula used to describe the relationship between the chemical reaction rate constant *k* and the ambient temperature. Their analysis revealed an equation in the form [20]

(2) $$k=A\cdot e^{-\frac{E_{a}}{R\cdot T}},$$

where, the *A* is the pre-exponential factor, which is related to the moisture content inside the polymer insulation material. *R* is a gas constant, and *R* = 8.314 J/mol·K. *T* is the temperature during the chemical reaction process. The time–temperature superposition theory [27,28] indicates that the effect generated by increasing temperature and prolonging the reaction duration on the molecular motion is equivalent. In other words, the variation law of the microstructure parameters of polymer insulation under high temperature can be observed under other conditions (both a lower temperature and longer duration). Thus, the FDS curve under the high temperature can be obtained by moving the curves under low temperature according to a shift factor, and the calculation equation is shown in Equation (3).

(3) $$\alpha_{T}=A\cdot e^{-\frac{E_{a}}{R\cdot T}}/A\cdot e^{-\frac{E_{a}}{R\cdot Tref}}=e^{\frac{E_{a}}{R}(\frac{1}{Tref}-\frac{1}{T})}$$

**Figure 6:**
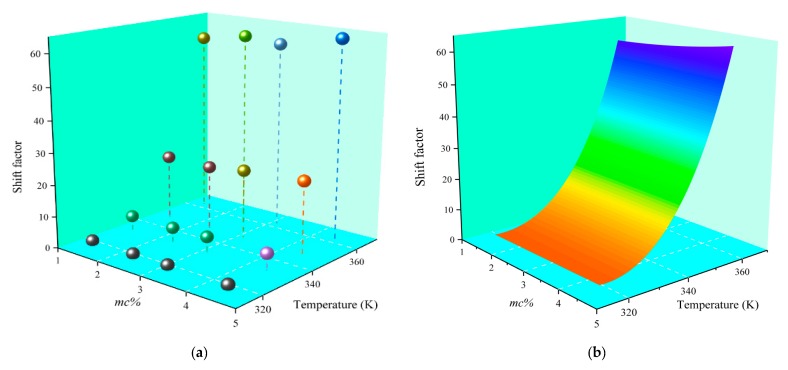
The variation law of shift factor. (**a**) The scatter plot of test temperature, *mc%* and shift factor; (**b**) The fitting surface of the shift factor.

From Equation (3), it is estimated that the value of the shift factor value is positively correlated with the activation energy and the test temperature. It has been shown in another published study that the value of the activation energy is related to the chemical reaction temperature and the concentration of the reactants [24]. It is worth mentioning that, as for the special operating environment of the transformer insulation system, the dominant reaction mechanism of the polymer aging should be hydrolysis when referring to the Arrhenius equation [24,29,30]. Therefore, once the synergy effect produced by moisture content and test temperature is ignored, the accuracy and versatility of the established model will be severely limited. In order to explore the variation rule of the shift factor under the synergy effect, in this article, the temperature, moisture content, and shift factor (shown in Table 2) are set to *x*, *y*, and *z* values, respectively, and their distribution in three-dimensional coordinates is plotted in Figure 6a.

It can be seen from Figure 6a, the value of the shift factor shows a regular variation trend with the altered moisture content and test temperature. In order to verify this viewpoint, the variation law shown in Figure 6 is studied so as to establish a functional model for predicting the shift factor under any test temperature and moisture content, where the fitting surface is shown in Figure 6b. From Figure 6b, each point on the fitting surface represents a shift factor under the clear moisture content and test temperature. Provided that the test temperature and moisture content of samples are known, its shift factor can be predicted by using the equation shown in Table 3.

In the equation in Table 3, the *x*, *y* represent the moisture content and test temperature, respectively. Table 3 shows that the residual sum of squares (i.e., Reduced Chi-Sqr) of the fitting equation is less than 1, and the fitting goodness (i.e., R-Square) is close to 1. Therefore, the results of mathematical statistics preliminarily prove the feasibility of the proposed equation. Further, the shift factor predicted by the universal equation is shown in Table 4. In summary, the present findings reveal that the shift factor corresponding to test temperature and moisture content can be accurately predicted by the reported universal approach (shown in Table 3).

## 5. A Scheme for Temperature Correction on FDS Curve Using Reported Universal Approach

This article aims to introduce a scheme for temperature correction on FDS curve so as to verify the feasibility and accuracy of the reported universal approach. Therefore, it is worth making the following interpretations. (1) The shift factor can be accurately predicted by using the universal model shown in Table 3. (2) The shifted FDS curves at different test temperatures were obtained by shifting the measured curves (at 45 °C) along the frequency axis according to a predicted shift factor. (3) Provided that the reported method is valid, the shifted FDS curves at any test temperature should coincide with the measured curves at 45 °C.

Further, several pressboards were selected as the study objects in this article so as to construct the FDS curve at the various test temperatures. In order to emphasize its universality, several pressboards with different insulation conditions (*mc%* and DP) were utilized for feasibility verification of the proposed approach, where, the aged pressboard with various DP is obtained by the process of accelerated thermal aging at 130 °C, and where the aging rate of the pressboard has been studied previously, predicting the variation law between the DP value (aging degree) and aging duration [24]. The pressboard samples designated as 1, 2, 3, and 4 are common transformer pressboards with type II, and prepared as described in a previous study [20]; the other T_4_ transformer pressboards with type I, and newly prepared in this work, are designated 5 and 6. The details of the above various pressboards are shown in Table 5.

**Figure 7:**
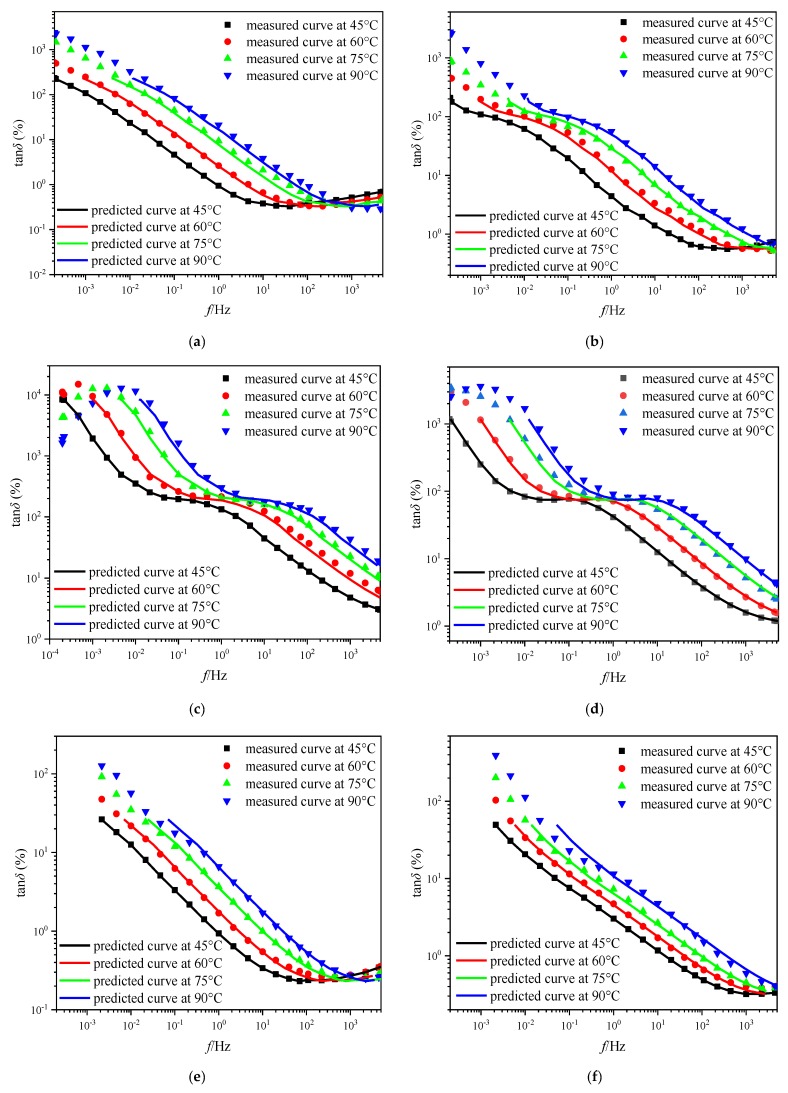
The verification test of the reported universal approach for temperature correction. (**a**) DP = 425, *mc%* = 1.2%, (**b**) DP = 994, *mc%* = 2.8%, (**c**) DP = 841, *mc%* = 3.7%, (**d**) DP = 1285, *mc%* = 4.0%, (**e**) DP = 663, *mc%* = 0.9%, (**f**) DP = 640, *mc%* = 1.3%.

Figure 7 presents the comparison of the shifted curve and measured curve at different temperatures. Obviously, the shifted curve obtained by the reported approach in this paper is basically coincident with the measured curve at any test temperature. Therefore, it is considered that the reported temperature correction method is effective. The obtained findings reveal that the reported approach is not only suitable for temperature correction on FDS curve of pressboard with different insulation conditions (*mc%* and degree of polymerization), but also maintains considerable accuracy when applied to different types of paperboard. The available study points out that the shift factor seems more likely temperature-dependent and moisture-dependent than aging-dependent [23]. The above conclusion is preliminarily confirmed in this work and described in Figure 6 and Figure 7. In summary, the experimental results of the temperature correction provide a basis for the feasibility verification of the proposed universal approach.

## 6. Conclusions

In order to correct the temperature effect on FDS curve of transformer polymer insulation, the knowledge of the shift factor is rather crucial. Therefore, a universal approach for temperature correction on FDS curve is reported. The present analysis and findings have led to the following conclusions.

- The reported approach for predicting the shift factor is based upon the depth analysis of the variation law among the shift factor, moisture content and test temperature. The findings reveal that the shift factor is both temperature-dependent and moisture-dependent.
- It is estimated that the value of the shift factor is positively correlated with the moisture content and test temperature. Moreover, the temperature correction results indicate that the shift factor mainly depends on the test temperature and moisture content rather than the aging degree.
- The verification results preliminarily reveal that the reported approach is not only suitable for temperature correction on FDS curve of pressboard with different insulation content, but also maintains considerable accuracy when applied to different types of paperboard. Therefore, the obtained conclusions will provide a universal idea for temperature correction on FDS curve of transformer polymer insulation.

## Figures and Tables

**Table 1:** The comparison of the parameters of two pressboards.

Pressboard Types	Type I	Type II
Brand	T_4_ transformer pressboard	Common transformer pressboard
Manufacturer	Taizhou Weidmann High Voltage Insulation Co., Ltd.	Chongqing AEA Group Transformer Co., Ltd.
Thickness	0.5 mm	2 mm
Tensile strength	MD: 98 MPa, CMD: 47 MPa	MD: 150.04 MPa, CMD: 57.14 MPa
Density	0.96 g/cm^3^	1.17 g/cm^3^

**Table 2:** The extracted shift factor of unaged pressboard with type I at different temperature.

No.	*mc%*	Measured Value			
		318.15 K	333.15 K	348.15 K	363.15 K
1	1.3%	1.00	4.43	20.45	57.79
2	2.3%	1.00	4.51	20.33	60.23
3	3.1%	1.00	4.84	21.83	59.14
4	4.4%	1.00	5.17	22.98	63.25

**Table 3:** The universal equation for predicting the shift factor.

$\alpha_{T}=\frac{Z_{0}+A_{01}\cdot x+B_{01}\cdot y+B_{02}\cdot y^{2}+C_{02}\cdot x\cdot y}{1+A_{1}\cdot x+B_{1}\cdot y+A_{2}\cdot x^{2}+B_{2}\cdot y^{2}+C_{2}\cdot x\cdot y}$					
*Z*_0_	−342,488	*A*_1_	67.5388	Precision	10^−15^
*A*_01_	−59.000	*A*_2_	0.55934	Degree of freedom	6
*B*_01_	2137.2	*A*_3_	−2.82072	Reduced Chi-Sqr	0.5229
*B*_02_	−3.3360	*B*_1_	0.00694	R-Square	0.9991
*C*_02_	−0.1576	*B*_2_	−0.18727	Fit Status	succeeded

**Table 4:** The predicted shift factor of unaged pressboard with type I.

No.	*mc%*	Predicted Value			
		318.15 K	333.15 K	348.15 K	363.15 K
1	1.3%	0.983	4.439	20.210	58.185
2	2.3%	0.988	4.603	20.847	59.013
3	3.1%	0.998	4.772	21.522	60.154
4	4.4%	1.027	5.139	23.013	63.056

**Table 5:** The predicted shift factor of various pressboards.

No.	DP	*mc%*	318.15 K	333.15 K	348.15 K	363.15 K
1	425	1.2%	0.983	4.428	20.158	58.137
2	994	2.8%	0.994	4.705	21.250	59.673
3	841	3.7%	1.010	4.926	22.143	61.318
4	1285	4.0%	1.016	5.012	22.495	62.010
5	663	0.9%	0.983	4.385	20.012	58.028
6	640	1.3%	0.983	4.439	20.210	58.185
